# Improving Bioinformatics Prediction of microRNA Targets by Ranks Aggregation

**DOI:** 10.3389/fgene.2019.01330

**Published:** 2020-01-28

**Authors:** Aurélien Quillet, Chadi Saad, Gaëtan Ferry, Youssef Anouar, Nicolas Vergne, Thierry Lecroq, Christophe Dubessy

**Affiliations:** ^1^ Normandie Univ, UNIROUEN, INSERM, Laboratoire Différenciation et Communication Neuronale et Neuroendocrine, Rouen, France; ^2^ Normandie Univ, UNIROUEN, UNIHAVRE, INSA Rouen, Laboratoire d'Informatique du Traitement de l'Information et des Systèmes, Rouen, France; ^3^ Normandie Univ, UNIROUEN, CNRS, Laboratoire de Mathématiques Raphaël Salem, Rouen, France

**Keywords:** microRNA, prediction, target mRNA, aggregation, database, receiver operating characteristic, precision and recall, F-score

## Abstract

microRNAs are noncoding RNAs which downregulate a large number of target mRNAs and modulate cell activity. Despite continued progress, bioinformatics prediction of microRNA targets remains a challenge since available software still suffer from a lack of accuracy and sensitivity. Moreover, these tools show fairly inconsistent results from one another. Thus, in an attempt to circumvent these difficulties, we aggregated all human results of four important prediction algorithms (miRanda, PITA, SVmicrO, and TargetScan) showing additional characteristics in order to rerank them into a single list. Instead of deciding which prediction tool to use, our method clearly helps biologists getting the best microRNA target predictions from all aggregated databases. The resulting database is freely available through a webtool called miRabel^1^ which can take either a list of miRNAs, genes, or signaling pathways as search inputs. Receiver operating characteristic curves and precision-recall curves analysis carried out using experimentally validated data and very large data sets show that miRabel significantly improves the prediction of miRNA targets compared to the four algorithms used separately. Moreover, using the same analytical methods, miRabel shows significantly better predictions than other popular algorithms such as MBSTAR, miRWalk, ExprTarget and miRMap. Interestingly, an F-score analysis revealed that miRabel also significantly improves the relevance of the top results. The aggregation of results from different databases is therefore a powerful and generalizable approach to many other species to improve miRNA target predictions. Thus, miRabel is an efficient tool to guide biologists in their search for miRNA targets and integrate them into a biological context.

## Introduction

Mature microRNAs (miRNAs) are small endogenous noncoding single strand RNAs. They regulate gene expression in eukaryotic organisms at the posttranscriptional level. Since their discovery in 1993 (Lee et al., 1993), it has been clearly established that miRNAs act as key regulators of several cell processes such as proliferation, differentiation, metabolism, and apoptosis (Krol et al., 2010; Shenoy and Blelloch, 2014); it is therefore not surprising to find them involved in numerous pathophysiological processes (Qu et al., 2014; Hommers et al., 2015; Reddy, 2015). To date, 2,654 mature human miRNAs are referenced in miRBase^2^ but several recent studies suggest that there may be a larger number (Friedlander et al., 2014; Jha et al., 2015; Londin et al., 2015; McCall et al., 2017). Each of them has the ability to potentially regulate several hundred of mRNAs and each targeted mRNA can be regulated by tens of miRNAs (Selbach et al., 2008; Friedman et al., 2009), thus creating a large and complex regulation network of gene expression unsuspected before. The bioinformatics identification of miRNA targets remains a challenge because mammalian miRNAs are characterized by a poor homology toward their target sequence except in the conserved “seed” region that comprises nucleotides 2–7 of the miRNA (Shin et al., 2010; Bartel, 2018). Nevertheless, several algorithms have been developed to include a set of features known to modulate the interaction between miRNA and their cognate mRNA in addition to the essential Watson-Crick pairings (Peterson et al., 2014). Among them, the most relevant are the free energy of the miRNA::mRNA system (Yue et al., 2009), the conservation of sequences among species (Brennecke et al., 2005) and the accessibility of binding sites (Long et al., 2007). This resulted in the creation of more than 187 target prediction tools (as of September 2019, from OMICtools' database (Henry et al., 2014)), all of which have their strengths and weaknesses (Marbach et al., 2012; Le et al., 2015). These tools are useful to reduce the number of potential targets in order to streamline the experimental validations (Witkos et al., 2011). However, their predictions suffer from a poor accuracy and sensitivity as revealed by experimental data (Thomas et al., 2010; Pinzon et al., 2017) and are very divergent from one tool to another (Min and Yoon, 2010). So far, no single method consistently outperforms others, thus supporting the idea that databases content combination is an efficient way to improve prediction relevance. Assuming that an interaction predicted by more than one algorithm is more likely to be functional, databases such as miRWalk (Dweep et al., 2011; Dweep and Gretz, 2015; Sticht et al., 2018), miRSystem (Lu et al., 2012), miRGator (Nam et al., 2008) or, more recently, Tools4miRs (Lukasik et al., 2016), store and/or compare results predicted by several popular tools using statistics and mRNA/protein expression data. Interestingly, it has been demonstrated that targets resulting from the intersection of two lists of predictions are not more likely to be present in the intersection of two other lists (Ritchie et al., 2009). Therefore, intersecting results does not increase the probability of retaining true positives and it may lead to decreased sensitivity because of possibly omitting valid interactions (Sethupathy et al., 2006; Oliveira et al., 2017). In order to circumvent these limitations, we computed a new score based on the aggregation of the interaction ranks taken from other well-known prediction algorithms. The goal being to give all available predicted interaction to the biologist for a given miRNA while highlighting the most relevant ones. To test our hypothesis, we aggregated four prediction algorithm results which enabled us to show that this new score significantly improves miRNA targets prediction compared to other prediction tools. To allow a more comprehensive analysis, the results of this aggregation were eventually linked to their respective cellular pathways using KEGG database, and implemented in a web tool named miRabel.

## Materials and Methods

### Aggregated Databases

Computationally predicted human miRNA::mRNA interaction databases generated by miRanda (Betel et al., 2010), PITA (Kertesz et al., 2007), SVMicrO (Liu et al., 2010) and TargetScan (Agarwal et al., 2015) were used. These publicly available online algorithms have been chosen because each of them uses different and complementary features of miRNA::mRNA interactions such as seed match, interspecies conservation, free energy, site accessibility and target-site abundance (**Table S1**) (Peterson et al., 2014). Only precomputed data were used since this is what is mostly accessible online to biologists. The ranks of each predicted interaction retrieved from one or more of these databases have been aggregated using the R package RobustRankAggreg (RRA, v1.1) (Kolde et al., 2012) with R (v3.2.0). Briefly, this method normalizes ranks with the maximal value of 1. The selected function (Mean, Default, Geometric mean, Median, Min, Stuart) is then used for lists aggregation. Finally, a probabilistic model is used to makes the algorithm parameter free and robust to outliers, noise, and errors. Missing values are replaced by the maximum relative rank value. The new score resulting from the aggregation is used to rerank each interaction and indicates the significativity of the proposed rank in miRabel.

### Testing Data Sets

**Figure 1:**
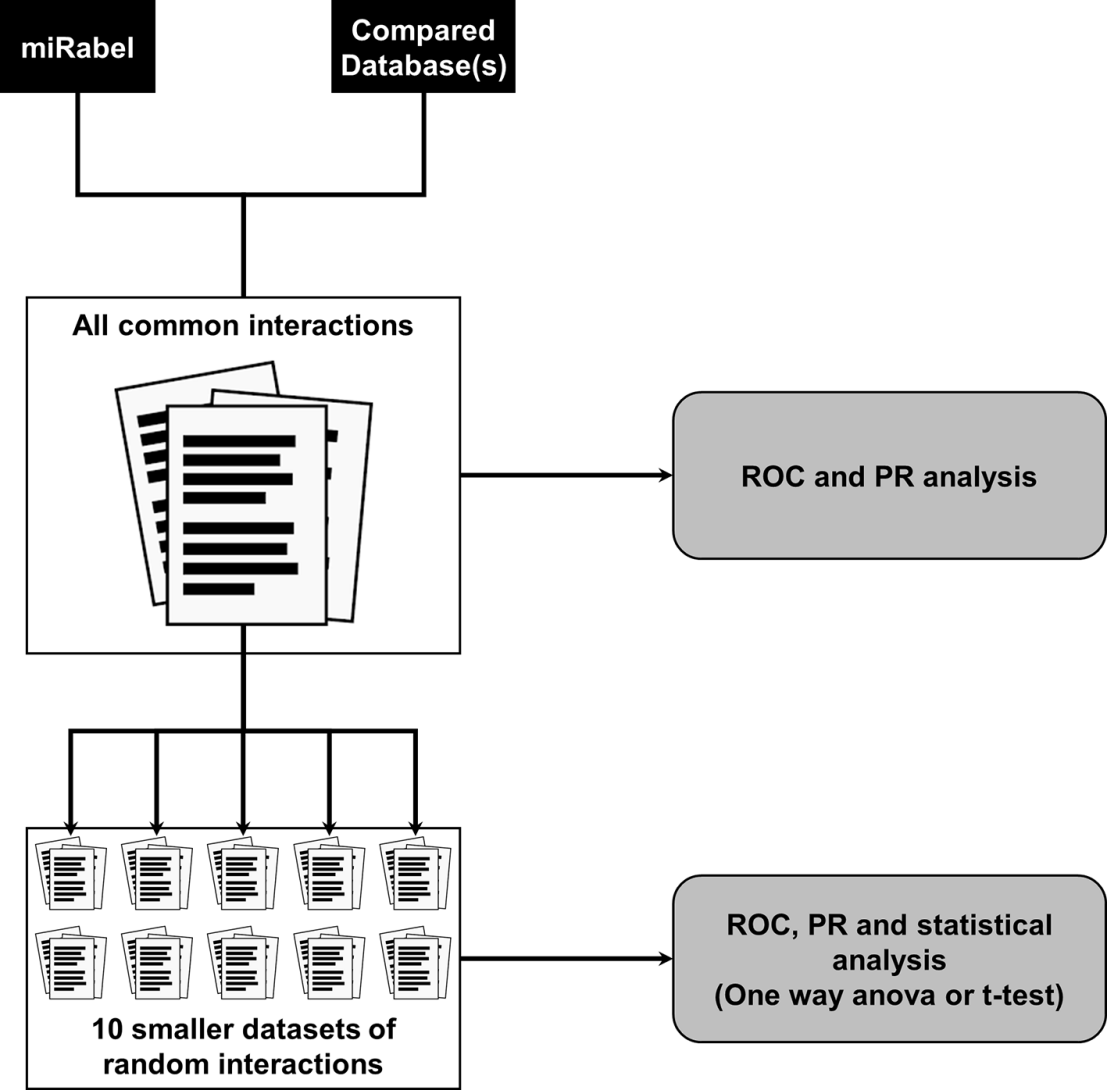
Testing data sets design and databases performance analysis methodology. A large data set containing all common interactions between compared databases is created. For ease of use, 10 smaller data sets of 50,000 interactions were randomly picked from all common ones. Predictions performance are then compared using receiver operating characteristic (ROC) and a precision and recall (PR) analysis on all data sets.

Two types of testing data sets were used for each of the comparisons described in this paper. First, to compare the different aggregation methods, we used one million randomly selected interactions within aggregated data. Validated interactions accounted for 5% of the testing data set. The use of all common interactions between compared databases resulted in extremely large data sets (>500,000 interactions) which reduced the amount of possible analysis due to computation time (several weeks). This led us to design a second type of data sets of 50,000 interactions randomly picked from the corresponding larger data set. For each large data set, 10 smaller ones were created (**Figure 1**). The amount of experimentally validated interactions within these randomly picked ones was set so as to remain close in proportion to the main, larger data set. These smaller data sets allowed us to increase the relevance and statistical significance of performance results.

### Performance Analysis Methods

On each data set, a receiver operating characteristic (ROC) analysis was done using the area under curve (ROC_AUC) as implemented in the R package pROC (Robin et al., 2011). To analyze top prediction results, a specificity of 90% was set as a threshold in order to compute partial ROC (pROC_90%_) and the corresponding AUC (ROC_pAUC_90%_) and sensitivity. To focus on which classifier better identifies true positive interactions, data sets were further compared with precision and recall (PR) curves using R programming as well. For the same purpose as with the pAUC of the ROC analysis, we calculated the harmonic mean between the precision and the recall (F-score) for different percentages of the top interactions.

### Statistics

Statistical analysis of results obtained with ten random data sets containing 50,000 interactions were done with R (version 3.4.0) using either a Repeated Measures One Way ANOVA with Dunnett's post-test or a Student t-test depending on the number of compared groups. Adjusted p-values are considered significant when <0.05.

## Results

### Mirabel Overview

#### miRabel: A Database for Microrna Target Predictions

**Figure 2:**
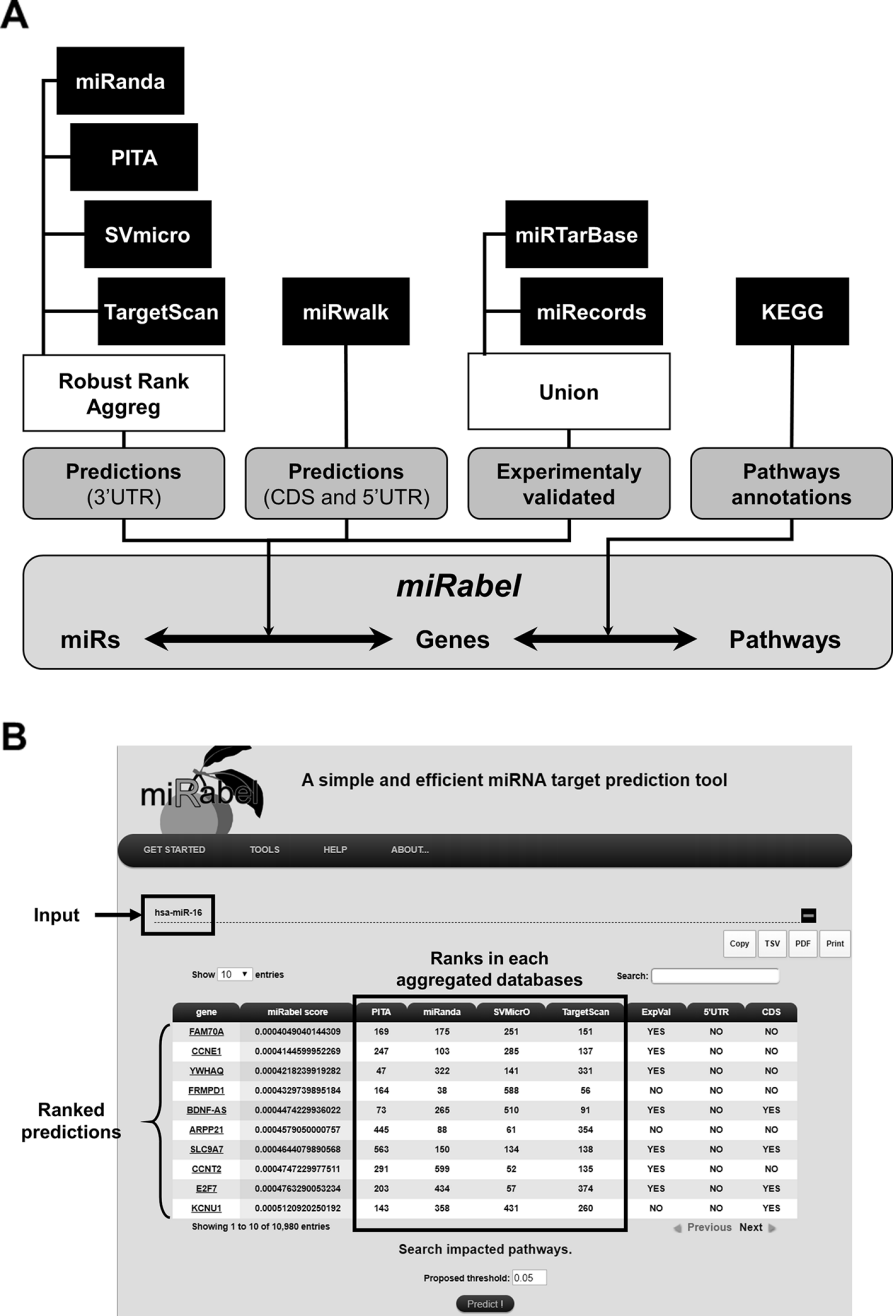
Overview of miRabel. Predictions results from miRanda, PITA, SVMicrO, and TargetScan for 3'UTR are aggregated using Robust Rank Aggreg. 5'UTR and CDS predictions are retrieved from miRWalk database. Experimentally validated interactions are identified using miRTarBase and miRecords. Links between predictions and pathways are established based on KEGG information **(A)**. An example of miRabel web interface is shown using predictions for hsa-miR-16. Predicted targets are ranked according to miRabel's score. Rank found for this interaction in each database are indicated as well as its experimental validation status and sub-localization in the mRNA (5'UTR and CDS) **(B)**.

The database was designed with MySQL^3^ using InnoDB motor and includes predictions from miRanda (Betel et al., 2010), PITA (v.6.0) (Kertesz et al., 2007), SVMicrO (Liu et al., 2010), and TargetScan (Agarwal et al., 2015). It contains tables for 2,587 human miRNAs which have target mRNAs, 19,799 genes and 275 pathways. This represents more than 14.7 million predicted interactions from which 351,298 are experimentally established. These experimentally validated interactions are annoted using miRTarBase (v.6.0) (Hsu et al., 2011) and miRecords (Xiao et al., 2009), whereas 5'UTR and CDS predictions were identified with miRWalk database (v.2.0) (Dweep et al., 2011). Genes and pathways information as well as their relationships were retrieved from KEGG's database while miRNA data were from miRBase (release 22.1) and linked with miRNA target predictions. Since the annotation of miRNAs has changed over the years, a tool was developed to convert the names of miRNA queries in the latest version used by miRBase. In order to standardize gene names from the different tools, they were converted to the NCBI gene ID and a table containing their synonyms has been built. Potential interactions between miRNAs and genes were obtained with our prediction method represented as shown in **Figure 2A**. Pathways linked to the resulting interactions can be retrieved and ranked according to the proportion of its interactions regulated by a given miRNA. For each pathway, the number of validated interactions for this miRNA is also indicated.

#### The Web Interface

The web interface was designed with PHP^4^ and CSS^5^. It enables users to query the system directly by miRNA, gene or pathway name (**Figure 2B**). Multiple queries are allowed in order to identify common prediction results. Queries by pathways are easily made thanks to asynchronous database queries and name completion. The results are visualized by using the DataTable plugin of the JQuery framework which allows to create tables that can be easily filtered and sorted. Results can be copied, printed or exported in tabulated-separated or pdf formats. An online documentation is also provided. miRabel is freely available^6^.

### Evaluating Aggregation Methods

**Figure 3:**
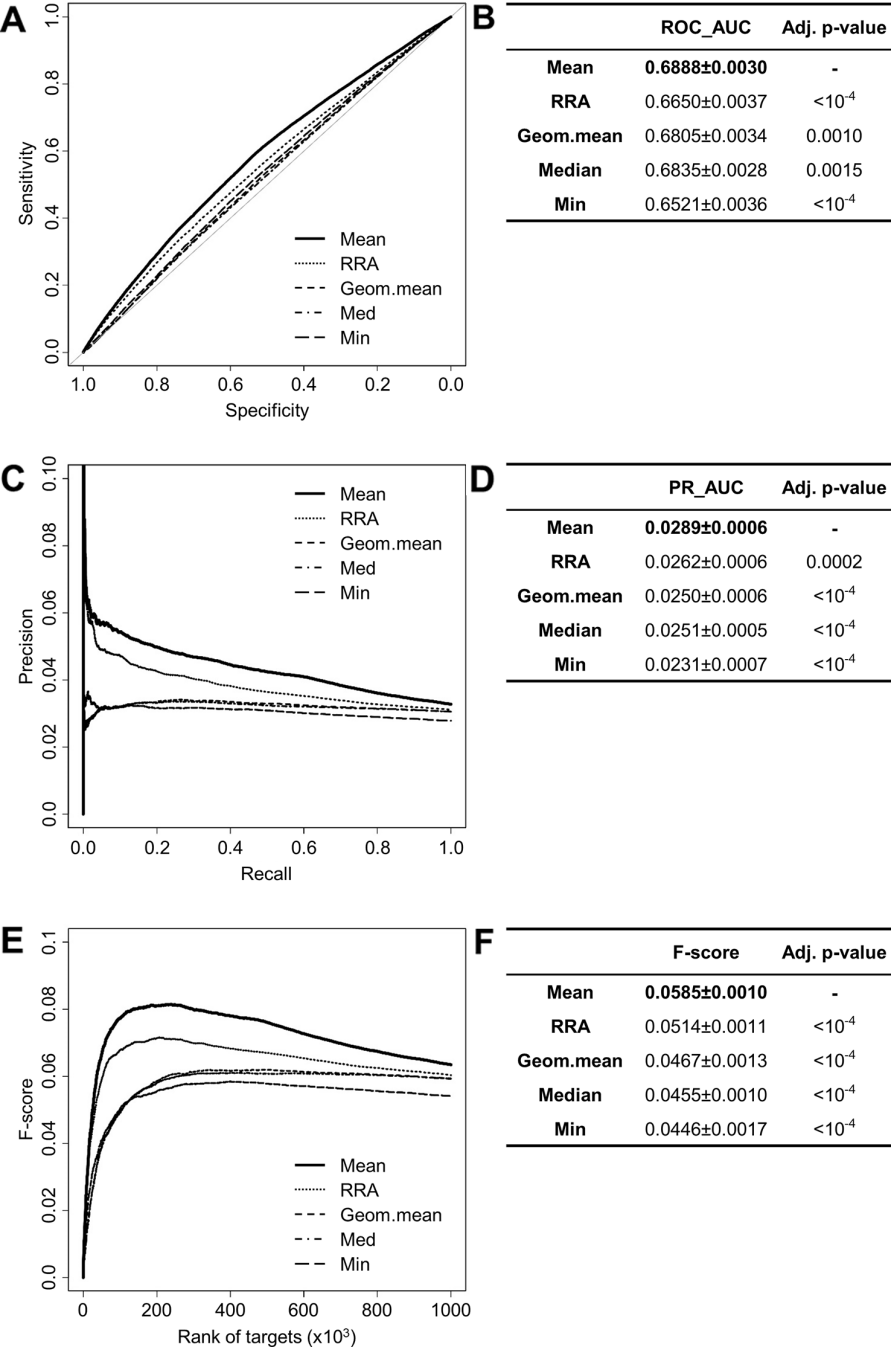
Performances comparison of aggregation methods. Receiver operating characteristic (ROC) curve analysis **(A)**, showing the sensitivity and the specificity for five aggregation methods from the RobustRankAggreg (RRA) R package, and their respective area under curves (AUC, **B**) have been calculated using the pROC R package on ten random data sets containing 50,000 interactions. Using the same data set, PR analysis **(C)** with PR_AUC **(D)** has been carried out. The cumulative harmonic mean between precision and recall (F-score) was also plotted **(E)** for each ranked interaction of this data set. The average F-score is reported for all interactions **(F)**. The higher are the ROC_AUC, PR_AUC and F-score, the better are the performances of the tested method. Highest values are in bold font.

The performances of the aggregation methods (Mean, Default, Geometric mean, Median, Min, Stuart) provided by RRA have been compared to each other (except for the Stuart method due to extensive computation time). ROC and PR analysis show that the mean of the ranks provides the best result (ROC_AUC_Mean_ = 0.6888 ± 0.0030, PR_AUC_Mean_ = 0.0289 ± 0.0006) (**Figures 3A–D**). Interestingly, the F-score for all interactions (F-score = 0.0585 ± 0.0010) indicates that the mean method is also the most consistent in promoting validated interactions (**Figures 3E, F**). When looking at top predictions only, the mean method remains significantly better than other compared methods (**Table S2**). These results led us to use the mean method to aggregate the ranks of miRanda, PITA, SVMicrO, and TargetScan which has been subsequently implemented in miRabel.

### Comparison to Aggregated Methods

**Figure 4:**
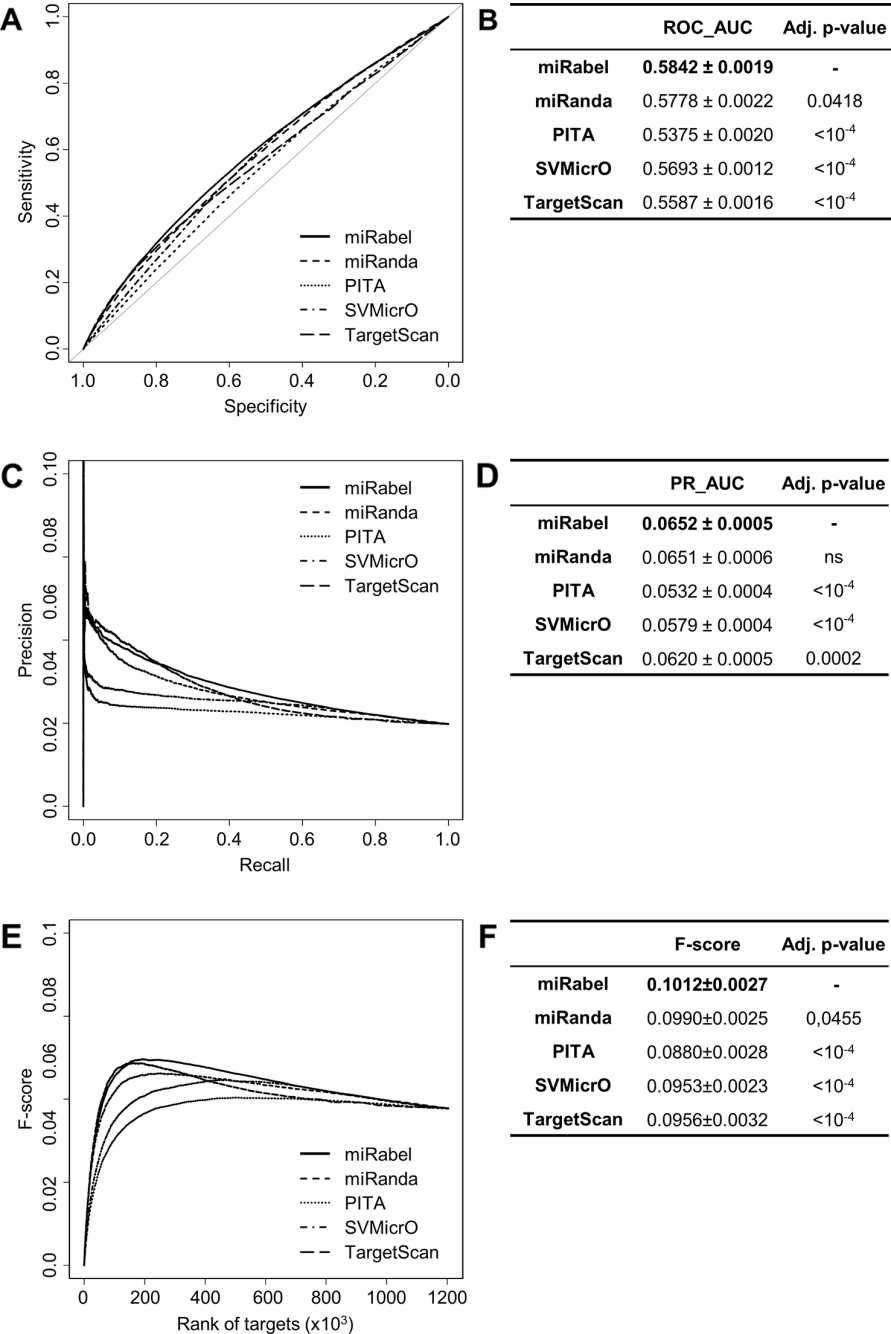
Performances comparison of aggregated prediction algorithms. Receiver operating characteristic (ROC) curve analysis **(A)**, showing the sensitivity and the specificity for miRabel, miRanda, PITA, SVMicrO, and TargetScan, and their respective AUC **(B)** have been calculated using the pROC R package on 1,204,591 common interactions. Using the same data set, a precision and recall (PR) analysis **(C)** with PR_AUC **(D)** has been carried. The cumulative harmonic mean between precision and recall (F-score) was also plotted **(E)** for each ranked interaction of this data set. The average F-score is reported for all interactions **(F)**. The higher are the ROC_AUC, PR_AUC and F-score, the better are the performances of the tested algorithm. Highest values are in bold font.

In order to test whether any improvement was gained with our aggregation method, the performances of each aggregated algorithms were compared to miRabel using ROC and PR analysis as well. These comparisons were done with 1,204,591 predicted interactions that are common to miRanda, PITA, SVMicrO, and TargetScan. Within these predictions, 59,743 are experimentally validated ones (**Figure 1**). ROC curve analysis shows that miRabel significantly improves the prediction of validated miRNA::mRNA interactions (ROC_AUC = 0.5842 ± 0.0019) compared to miRanda, PITA, SVMicrO, and TargetScan (**Figures 4A, B**). This improvement is even visuable with the PR analysis (PR_AUC = 0.0652 ± 0.0005) (**Figures 4C, D**) and the consistency of miRabel superior F-score throughout the data set (**Figures 4E, F**). A significant improvement was also manifest for the aggregated predictions for the top ranked interactions (ROC_pAUC_90%_ = 0.0096 ± 0.0001; F-score_20%_ = 0.1006±0.004) compared to PITA, SVMicrO and TargetScan (**Table S3**).

### Comparison to Other Prediction Tools

**Figure 5:**
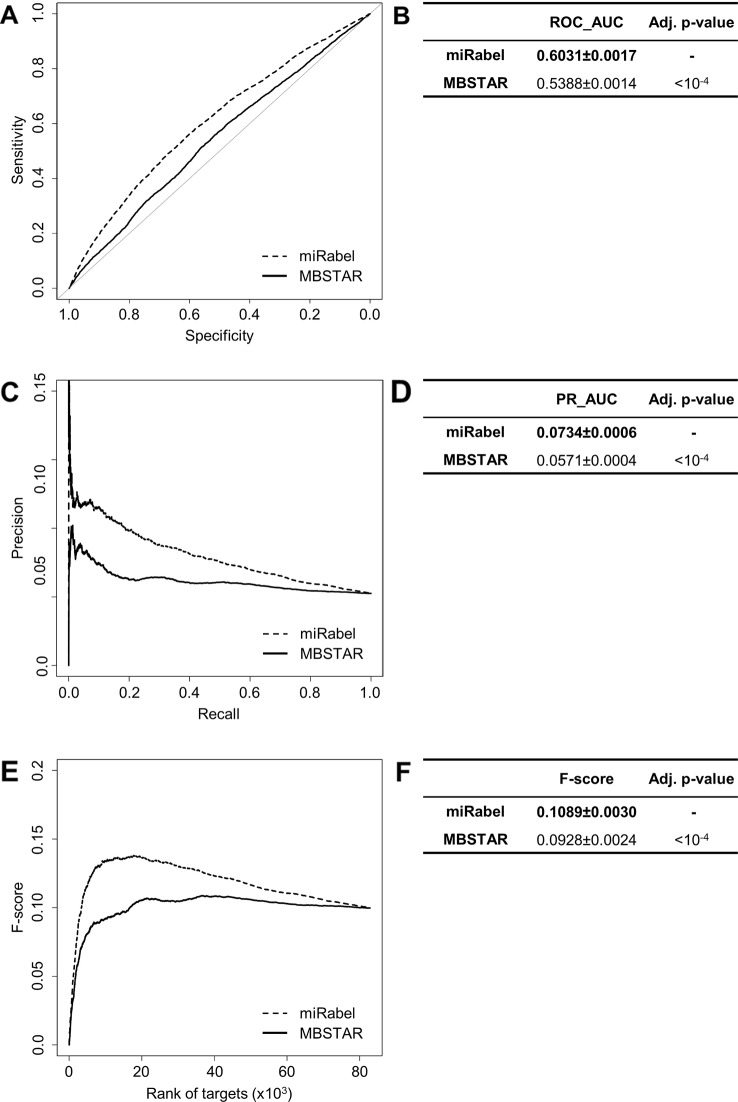
Performances comparison of miRabel and MBSTAR. Receiver operating characteristic (ROC) curve analysis **(A)**, showing the sensitivity and the specificity for miRabel and MBSTAR, and their respective area under curves (AUC) **(B)** have been calculated using the pROC R package on 82,867 common interactions. Using the same data set, a precision and recall (PR) analysis **(C)** with PR_AUC **(D)** has been carried out. The cumulative harmonic mean between precision and recall (F-score) was also plotted **(E)** for each ranked interaction of this data set. The average F-score is reported for all interactions **(F)**. The higher are the ROC_AUC, PR_AUC, and F-score, the better are the performances of the tested algorithm. Highest values are in bold font.

**Figure 6:**
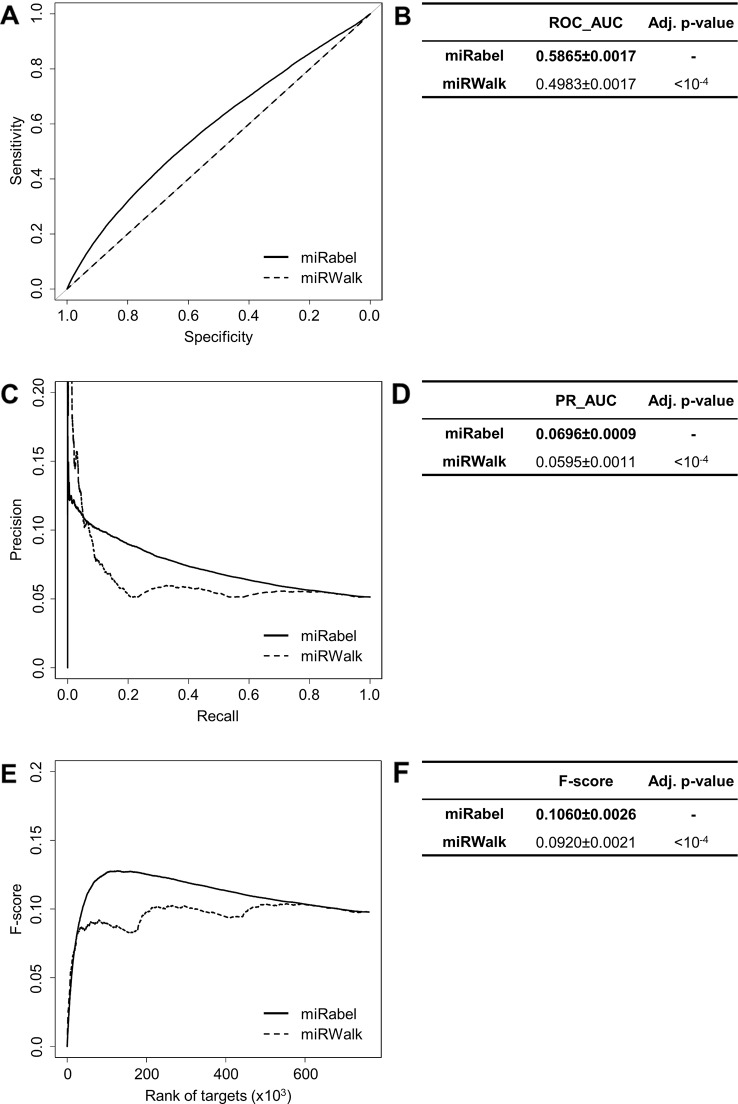
Performances comparison of miRabel and miRWalk. Receiver operating characteristic (ROC) curve analysis **(A)**, showing the sensitivity and the specificity for miRabel and miRWalk, and their respective area under curves (AUC) **(B)** have been calculated using the pROC R package on 761,354 common interactions. Using the same data set, a precision and recall (PR) analysis **(C)** with PR_AUC **(D)** has been carried out. The cumulative harmonic mean between precision and recall (F-score) was also plotted **(E)** for each ranked interaction of this data set. The average F-score is reported for all interactions **(F)**. The higher are the ROC_AUC, PR_AUC and F-score, the better are the performances of the tested algorithm. Highest values are in bold font.

**Figure 7:**
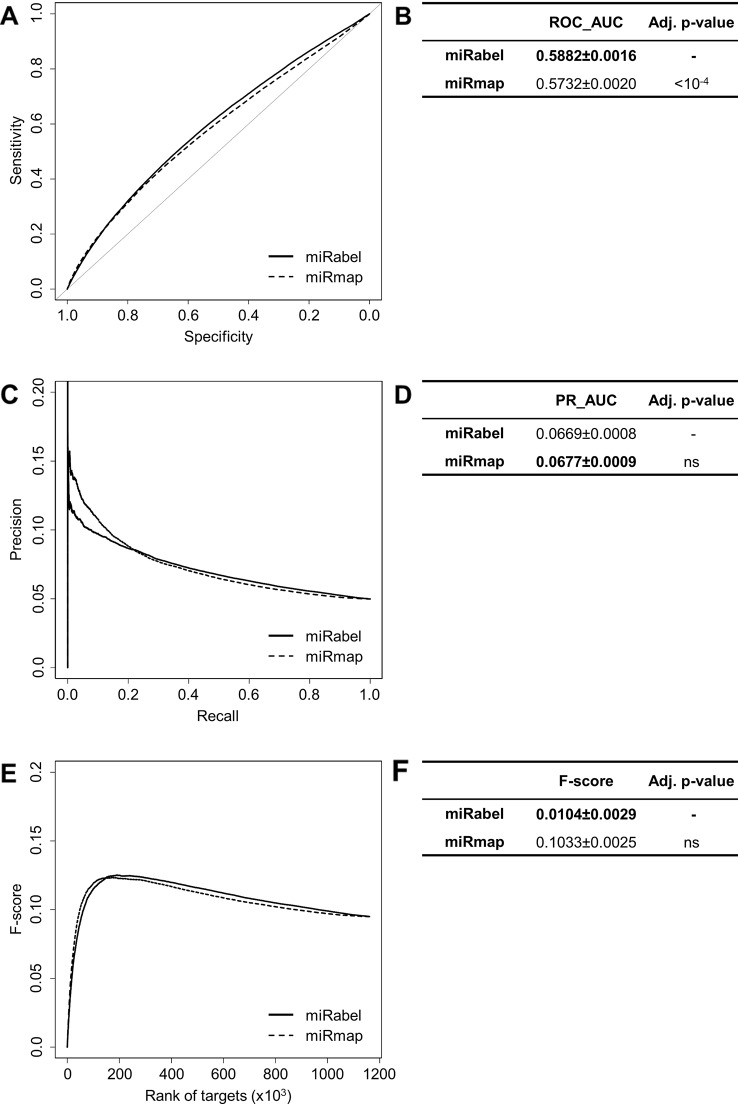
Performances comparison of miRabel and miRmap. Receiver operating characteristic (ROC) curve analysis **(A)**, showing the sensitivity and the specificity for miRabel and miRmap, and their respective area under curves (AUC) **(B)** have been calculated using the pROC R package on 1,160,781 common interactions. Using the same data set, a precision and recall (PR) analysis **(C)** with PR_AUC **(D)** has been carried out. The cumulative harmonic mean between precision and recall (F-score) was also plotted **(E)** for each ranked interaction of this data set. The average F-score is reported for all interactions **(F)**. The higher are the ROC_AUC, PR_AUC and F-score, the better are the performances of the tested algorithm. Highest values are in bold font.

**Figure 8:**
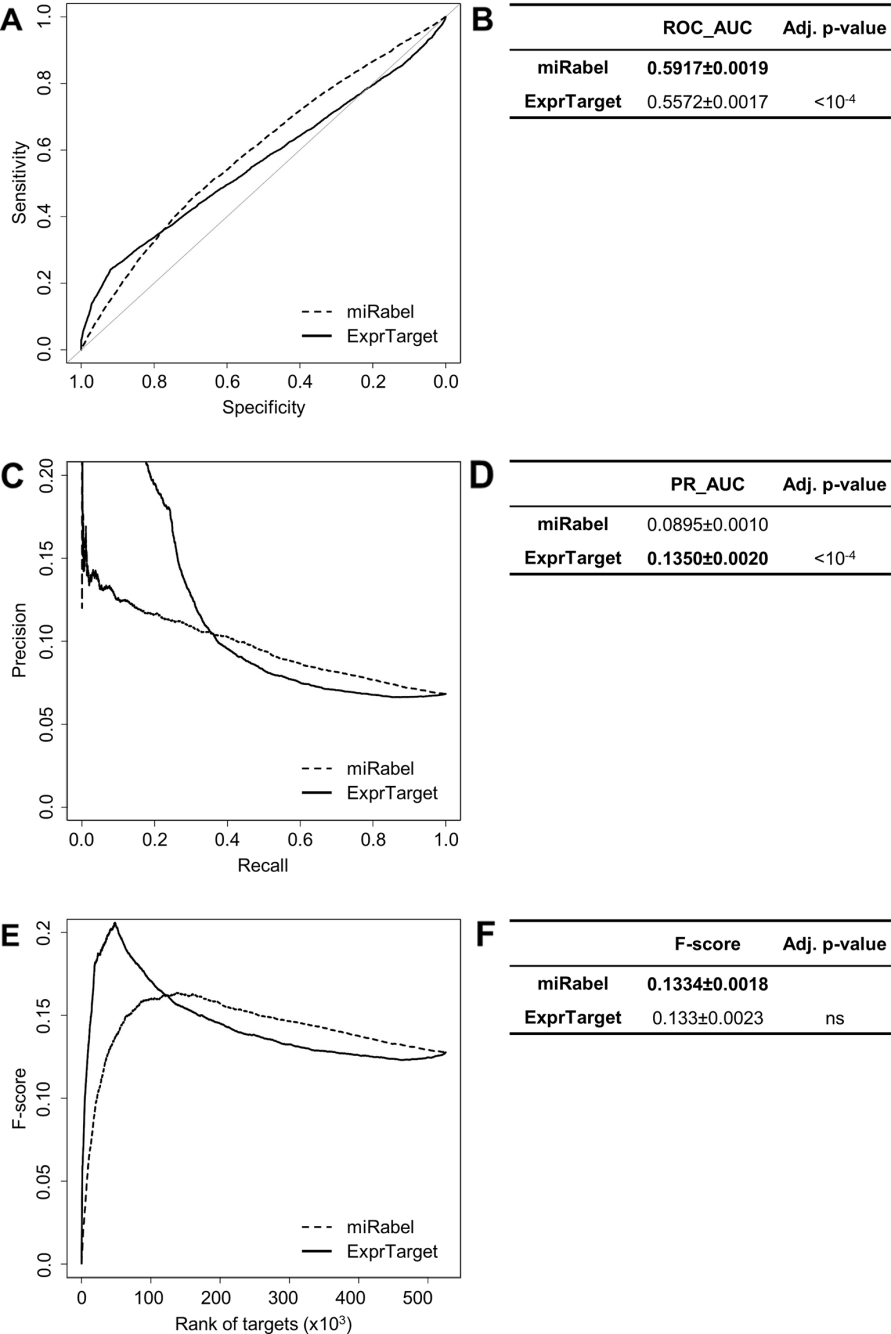
Performances comparison of miRabel and ExprTarget. Receiver operating characteristic (ROC) curve analysis **(A)**, showing the sensitivity and the specificity for miRabel and ExprTarget, and their respective area under curves (AUC) **(B)** have been calculated using the pROC R package on 105,122 common interactions. Using the same data set, a precision and recall (PR) analysis **(C)** with PR_AUC **(D)** has been carried out. The cumulative harmonic mean between precision and recall (F-score) was also plotted **(E)** for each ranked interaction of this data set. The average F-score is reported for all interactions **(F)**. The higher are the ROC_AUC, PR_AUC and F-score, the better are the performances of the tested algorithm. Highest values are in bold font.

Even though they are not aggregation methods, we also compared miRabel to four efficient, up-to-date and/or widely used prediction web tools (Fan and Kurgan, 2015): MBSTAR (Bandyopadhyay et al., 2015), miRWalk (v.2.0) (Dweep et al., 2011), miRmap (Vejnar and Zdobnov, 2012), and ExprTarget (Gamazon et al., 2010). ROC and PR curves analysis shows that our prediction data significantly improves the overall prediction of miRNA targets when compared to MBSTAR (**Figure 5** and **Table S4**) and miRWalk (**Figure 6** and **Table S5**). However, even though miRabel performs slightly better than miRmap (**Figures 7A, B**) and ExprTarget (**Figures 8A, B**), they seem fairly equal for true positives identification (**Figures 7C–F** and **8C–F**). Partial specificity and sensitivity (ROC_pAUC_90%_) of our aggregated data are also higher than those of MBSTAR (**Table S4**) and miRWalk (**Table S5**) whereas these parameters are significantly better for miRmap and ExprTarget (**Tables S6** and **S7**).

## Discussion

The prediction of miRNA targets is a bioinformatic challenge. Actually, each algorithm incorporates its own characteristics and the comparison of their results highlights important contradictions in their respective predictions (Shirdel et al., 2011; Fan and Kurgan, 2015). We therefore hypothesized that the aggregation of the predictions of several algorithms would improve the relevance and the robustness of the prediction of miRNA targets.

In order to validate this concept, we aggregated the predictions of four algorithms, miRanda, PITA, SVMicrO, and TargetScan, because they use different but complementary information. The results they provide are different both in terms of probability of interaction (i.e., their ranking) and of number of targets (Fan and Kurgan, 2015). Thus, only 8.1% of total interactions (1,204,591 / 14.7 million) are common to each other. The example of hsa-miR-16 (**Figure 2B**) also illustrates very well these divergences of predictions. Moreover, because some of these algorithms have not been updated recently, some more refined features found in recent prediction approaches such as TarPmiR (Ding et al., 2016), are not emphasized in our aggregated results if not at all present. Only human miRNAs were used initially to limit the amount of data as well as the associated computation times, but our approach is generalizable to miRNAs of all origins. Although there is so far a high prevalence of miRNA interaction sites found in the 3'UTR, recent papers have shown that some miRNAs can also regulate mRNAs by binding with the 5'UTR and CDS region of their targets (Moretti et al., 2010; Qu et al., 2016). Even though the value of these sites is not yet clearly established in the literature, this information remains important to get an integrated view of the predicted miRNA interaction sites on the mRNA. Since the score generated by the RRA package is also representative of the significativity of the ranking (the lower the score, the better) for a given interaction, we suggest to use miRabel with a threshold of 0.05. However, further analyses are required to really define an optimal threshold for miRabel. Finally, the choice of algorithms is also limited by the free availability of their database. To further improve predictions, it would therefore be interesting to include promising tools such as ComiR (Coronnello and Benos, 2013) whose prediction algorithm have been shown to perform well (Fan and Kurgan, 2015). RNAHybrid (Rehmsmeier et al., 2004) and rna22 (Miranda et al., 2006) are also of particular interest because they allow the prediction of targets in CDS and 5'UTRs and have been used successfully in predicting targets that were later validated experimentally.

We chose the RRA package for its ability to handle incomplete rankings and its robustness to noise due to divergent lists (Kolde et al., 2012). Comparing five of the aggregation methods included in the RRA package shows that the “mean” method is best for aggregating miRNA prediction lists (**Figure 3**, **Table S2**). However, although statistically significant, these values are relatively close to one another. These results are similar to those obtained in studies designed to compare the performance of several rank aggregation methods (Burkovski et al., 2012; Wald et al., 2012; Dittman et al., 2013). Among other aggregation methods, Cross Entropy Monte-Carlo has been found to be inadequate for our study due to too extensive computation times with large lists of items (Lin, 2010). Another method that could be evaluated is the Borda count algorithm (Dwork et al., 2001) which has already been used to aggregate cancer expression microarrays and proteomics data sets into a single optimal list (Jurman et al., 2008).

MiRabel performs better than each of the individual aggregated algorithms (**Figure 4**). Prediction improvement is also visible in the top ranked interactions (**Table S3**), thus showing that our aggregation moves validated interactions up in ranking. This is in line with multiple studies that combine data to obtain the most relevant interactions (Shirdel et al., 2011; Marbach et al., 2012; Friedman et al., 2014; Andres-Leon et al., 2015; Sedaghat et al., 2018). A recent study in particular shows that the union of the predictions of three tools among four (TargetScan, miRanda-mirSVR, RNA22) increases the performance of the analyses (Oliveira et al., 2017). However, our work goes further since prediction lists were aggregated and reranked in a unique list. The performance of their method was evaluated using only ten miRNAs and 1,400 genes but not the entire database. In order to avoid selection bias of the data sets, we analyzed all 1,204,591 interactions common to miRabel and the four aggregated algorithms, which represent 514 miRNAs and 15,343 genes. Furthermore, even though miRabel aggregates mostly older databases, it shows equal (vs. miRmap) or better (vs. MBSTAR and miRWalk) performances than up-to-date algorithms, thus clearly establishing that our method, even though simple, has a great potential. Interestingly, from all evaluations done with our data sets and methodology, we found other algorithm performances to be quite different from what was originally described in their respective original publications. This is in agreement with previous studies that highlighted the importance of testing prediction results on multiple, independent data sets and with a standardized evaluation protocol (Fan and Kurgan, 2015; Sedaghat et al., 2018). This is also one of the strengths of our study. Indeed, throughout all comparisons, miRabel was tested on 66 different data sets, which gives more robustness to the performance values calculated for our method.

As a conclusion, miRabel is a new efficient tool for the prediction of miRNA target mRNAs and their associated biological functions. Using an aggregation method, we improved the relevance of the predictions of three available algorithms. This promising approach can easily be extended to all publicly available databases or to other species. Moreover, the integrated biological pathways provide a more comprehensive view into the complex regulatory network of miRNAs. Eventually, there is no doubt that this method will greatly contribute in helping biologists gather all available predictions for a given miRNA and to highlight the most relevant potential interactions.

## Data Availability Statement

The datasets analyzed for this study can be found in the miRabel's database (http://bioinfo.univ-rouen.fr/mirabel/).

## Author Contributions

In this study, CD conceived the concept of the work and designed the study. AQ, CS, GF, and TL collected the data, developped the database and the miRabel's website. AQ and NV performed the data and the statistical analyses. AQ, NV, TL, and CD interpreted data. AQ and CD drafted the manuscript. AQ, TL, YA, and CD revised and finalized the manuscript. All authors read and approved the final manuscript.

## Funding

This work was supported by Ligue Contre le Cancer de Haute-Normandie (AAP2008 to YA and CD, AAP2017 to CD); Institut de Recherche et d'Innovation Biomédicale de Normandie (AAP2012 to YA and CD); Conseil Régional de Haute-Normandie, Institut National de la Santé et de la Recherche Médicale (UMRS1239); and the University of Rouen Normandy.

## Conflict of Interest

The authors declare that the research was conducted in the absence of any commercial or financial relationships that could be construed as a potential conflict of interest.

The reviewer RM and handling Editor declared their shared affiliation.

## Footnotes

2 http://www.mirbase.org/, release 22.1

3
http://www.mysql.com/

4
http://www.php.net

5
http://www.cssflow.com/

6
http://bioinfo.univ-rouen.fr/mirabel/

## Abbreviations

AUC, area under curve; PR, precision and recall; ROC, receiver operating characeristic.
