# Supplementary figures and images for: Improving Bioinformatics Prediction of microRNA Targets by Ranks Aggregation
Source: Front Genet. 2020 Jan 28;10:1330. doi: 10.3389/fgene.2019.01330 (PMC6997536; doi:10.3389/fgene.2019.01330)

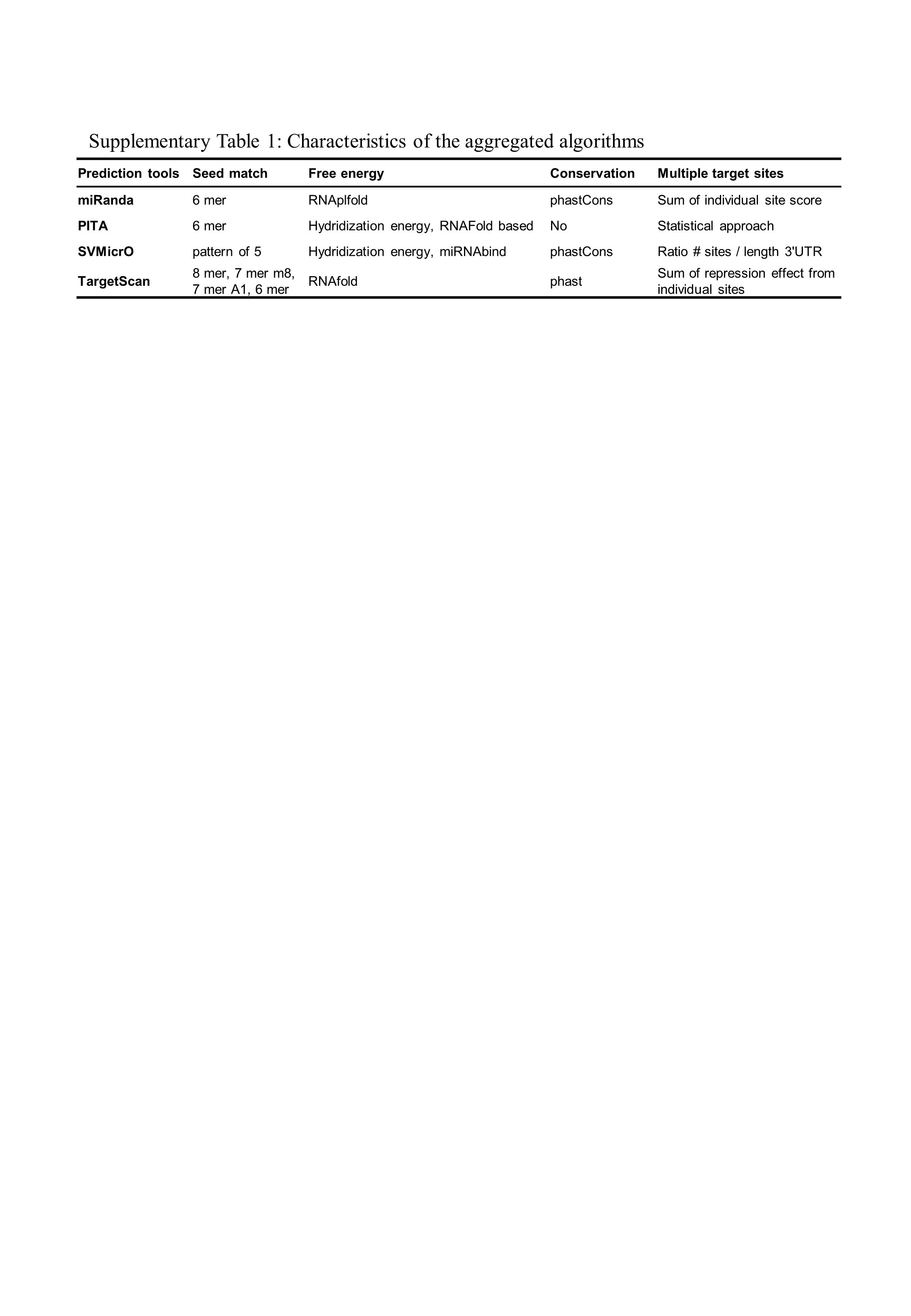

Supplement: Supplementary file 1 [file Image_1.tif]

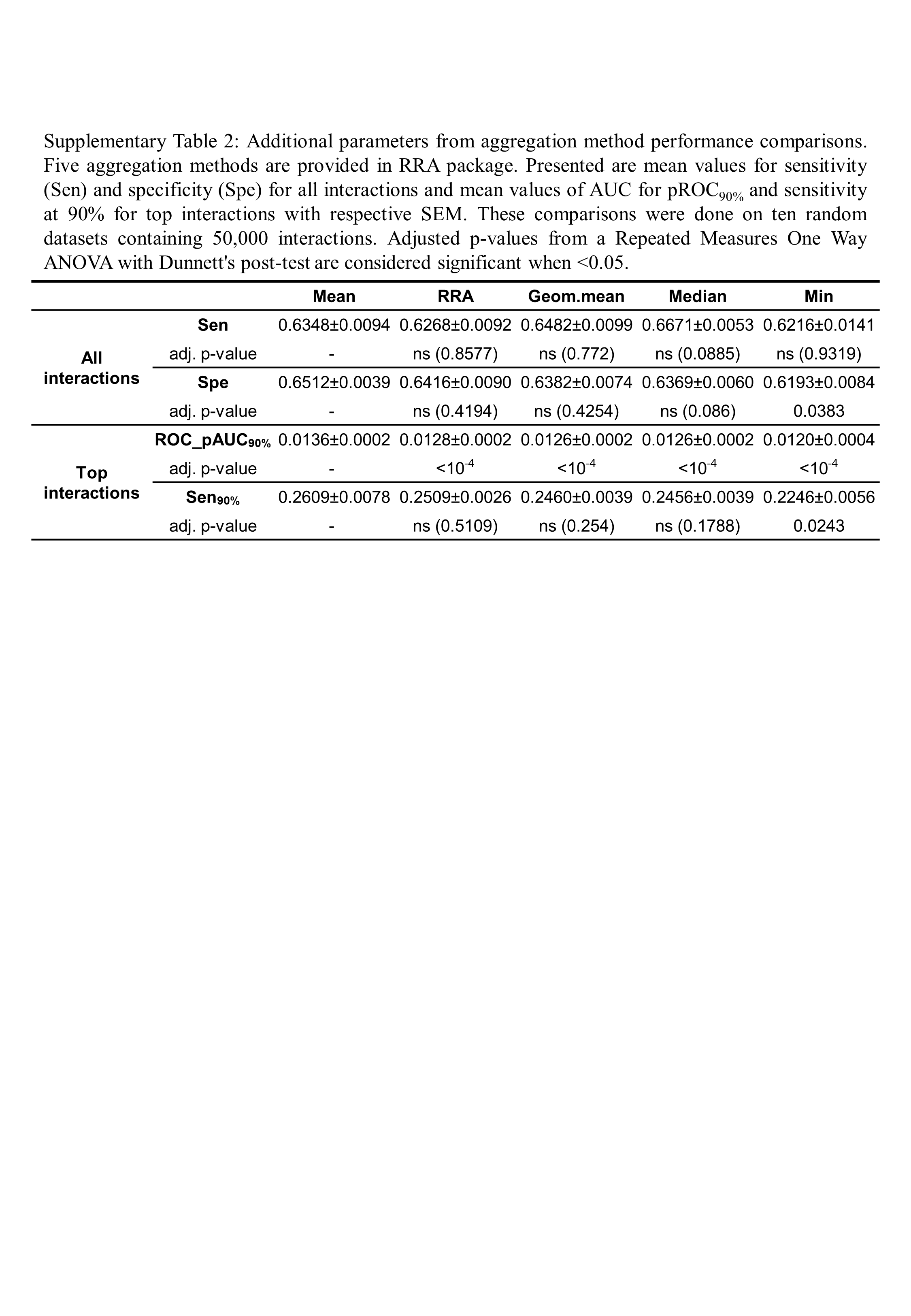

Supplement: Supplementary file 2 [file Image_2.tif]

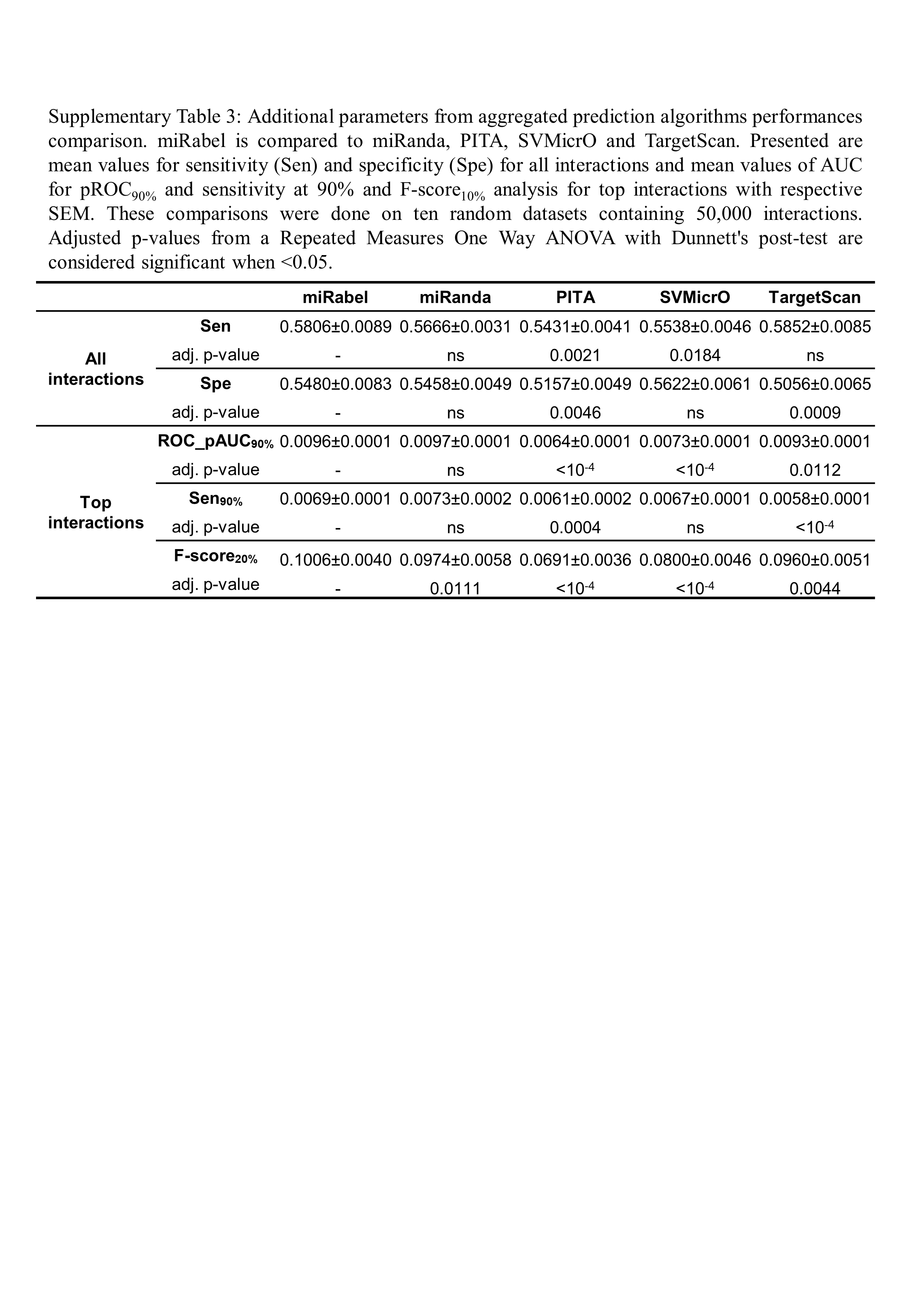

Supplement: Supplementary file 3 [file Image_3.tif]

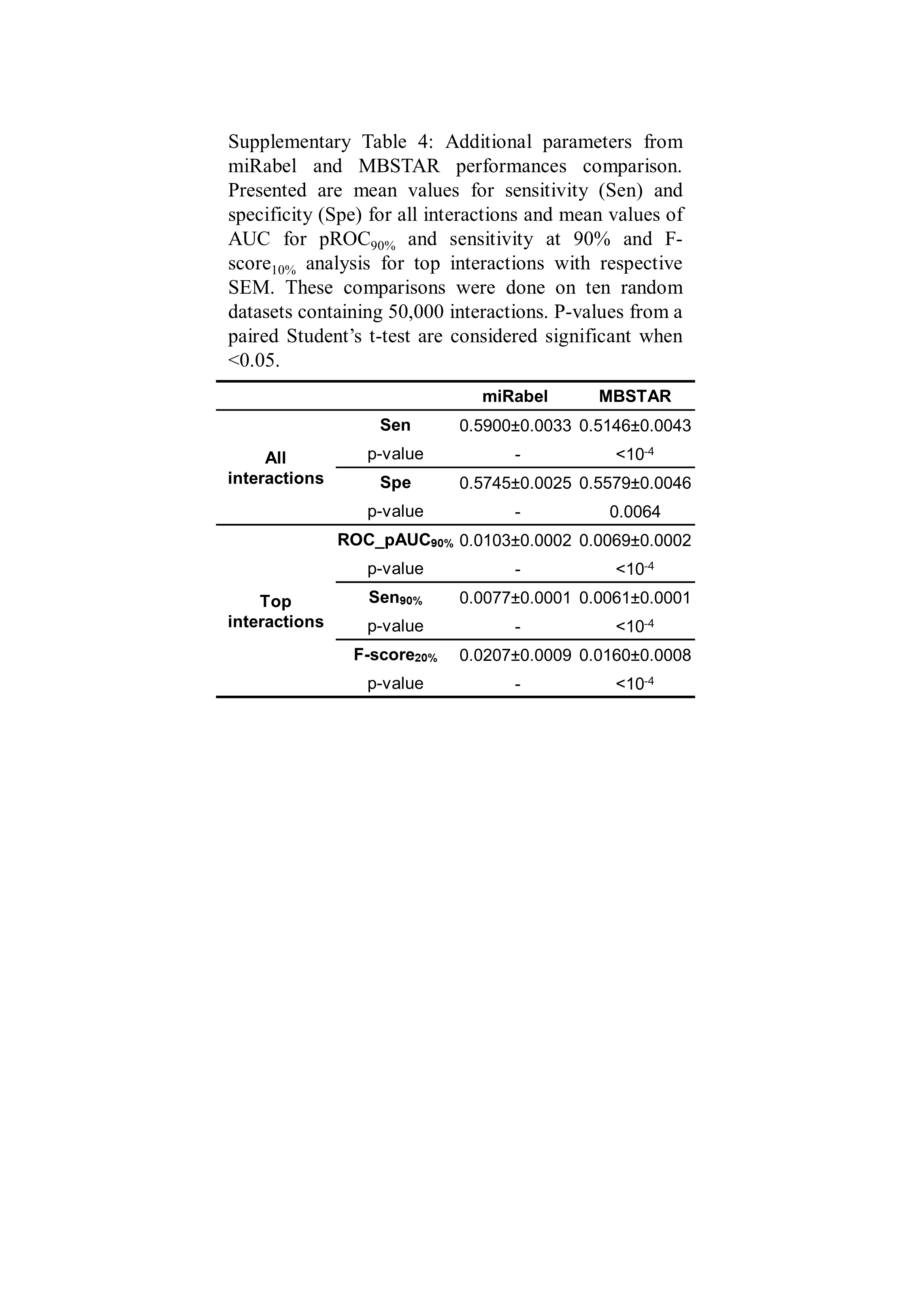

Supplement: Supplementary file 4 [file Image_4.tif]

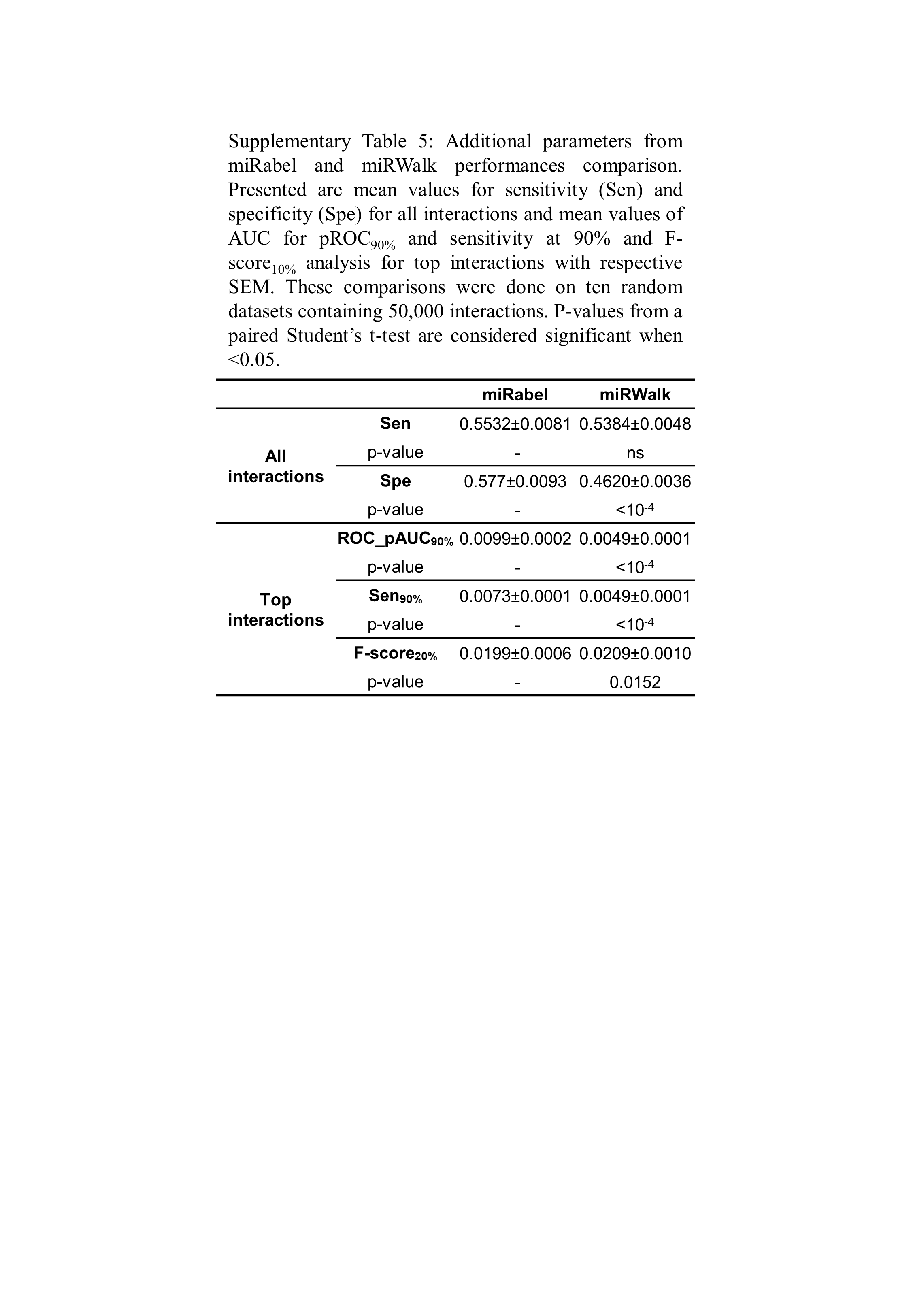

Supplement: Supplementary file 5 [file Image_5.tif]

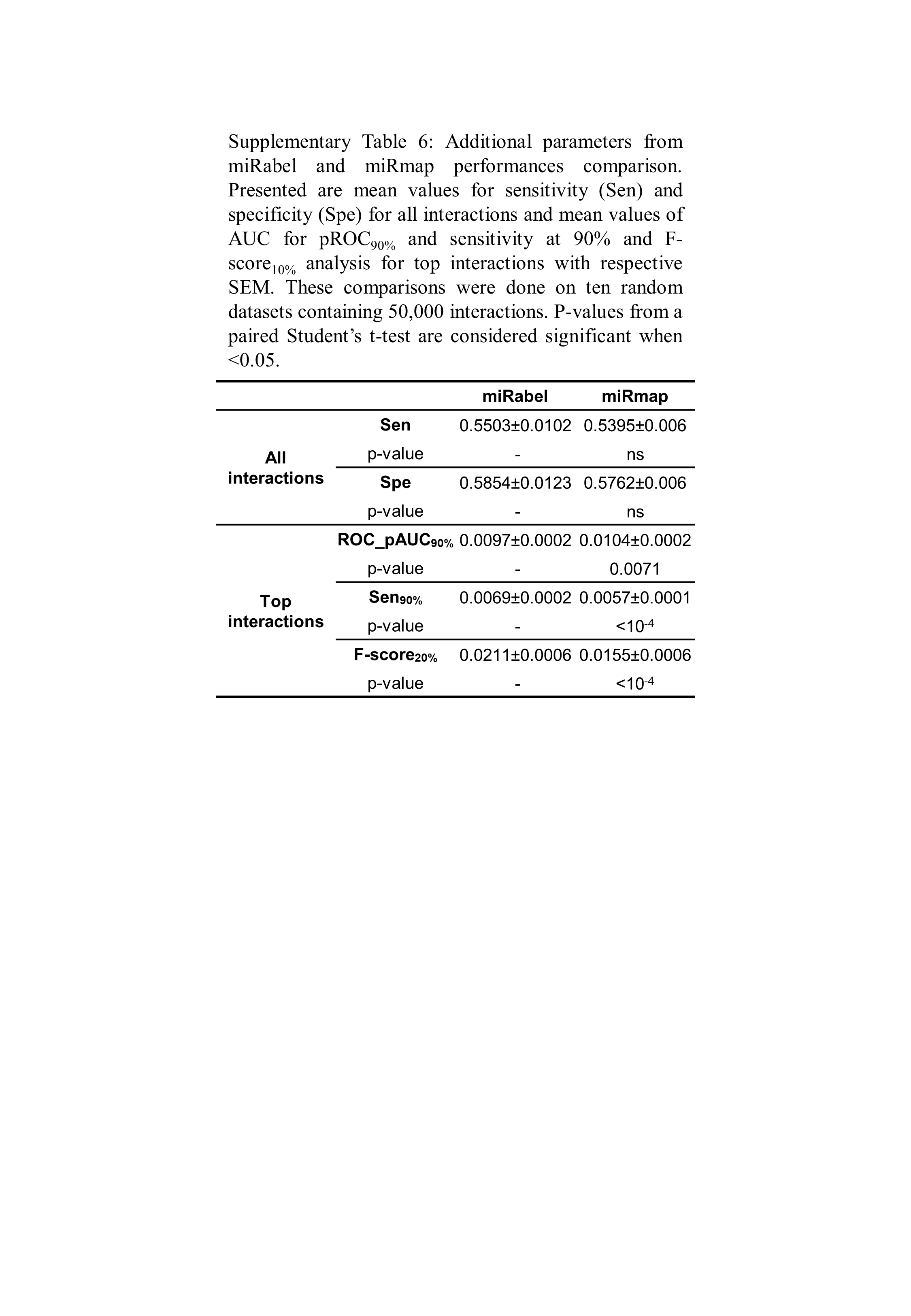

Supplement: Supplementary file 6 [file Image_6.tif]

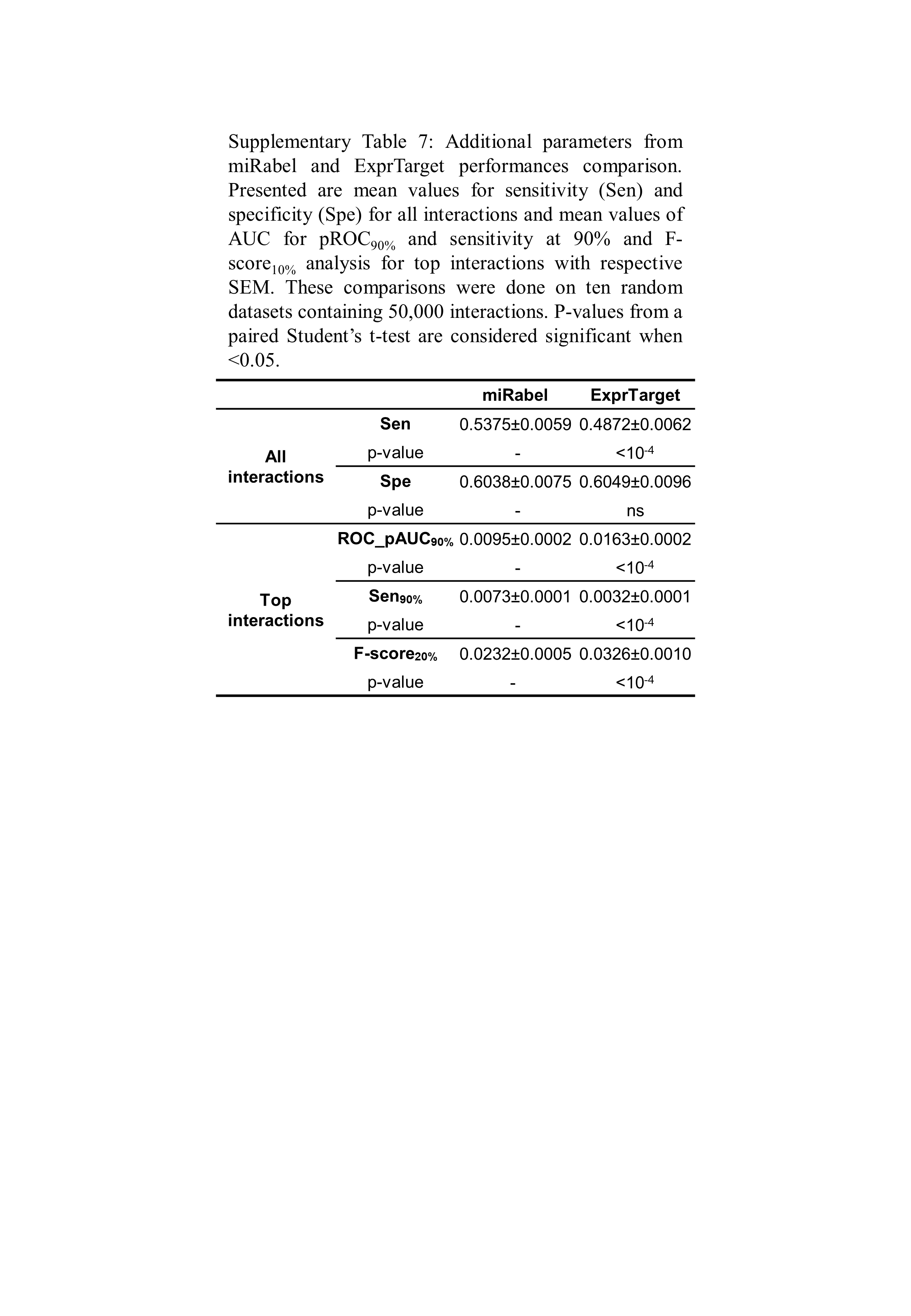

Supplement: Supplementary file 7 [file Image_7.tif]
